# PD-1 and Tim-3 Pathways Regulate CD8^+^ T Cells Function in Atherosclerosis

**DOI:** 10.1371/journal.pone.0128523

**Published:** 2015-06-02

**Authors:** Ming-Ke Qiu, Song-Cun Wang, Yu-Xin Dai, Shu-Qing Wang, Jing-Min Ou, Zhi-Wei Quan

**Affiliations:** 1 Department of General Surgery, Xinhua Hospital, Shanghai JiaoTong University, School of Medicine, Shanghai, China; 2 Laboratory for Reproductive Immunology, Hospital and Institute of Obstetrics and Gynecology, Fudan University Shanghai Medical College, Shanghai, China; University of Amsterdam Academic Medical Center, NETHERLANDS

## Abstract

T cell-mediated immunity plays a significant role in the development of atherosclerosis (AS). There is increasing evidence that CD8^+^ T cells are also involved in AS but their exact roles remain unclear. The inhibitory receptors programmed cell death-1 (PD-1) and T cell immunoglobulin and mucin domain 3 (Tim-3) are well known inhibitory molecules that play a crucial role in regulating CD8^+^ T cell activation or tolerance. Here, we demonstrate that the co-expression of PD-1 and Tim-3 on CD8^+^ T cells is up-regulated in AS patients. PD-1^+^ Tim-3^+^ CD8^+^ T cells are enriched for within the central T (T_CM_) cell subset, with high proliferative activity and CD127 expression. Co-expression of PD-1 and Tim-3 on CD8^+^ T cells is associated with increased anti-atherogenic cytokine production as well as decreased pro-atherogenic cytokine production. Blockade of PD-1 and Tim-3 results in a decrease of anti-atherogenic cytokine production by PD-1^+^ Tim-3^+^ CD8^+^ T cells and in an augmentation of TNF-α and IFN-γ production. These findings highlight the important role of the PD-1 and Tim-3 pathways in regulating CD8^+^ T cells function in human AS.

## Introduction

Atherosclerosis (AS), a chronic inflammatory disease [1], is considered to be responsible for a great number of deaths worldwide, in particular due to its association with coronary artery disease. Though hypercholesterolemia, hypertension, and smoking are thought to be the etiological factors of this disease, it is well established that chronic immune stimulation plays an important role in all stages of AS[2]. Monocytes and T cells migrate to the arterial tissue via chemokine/chemokine receptor interactions. Monocytes then differentiate into macrophages, accumulate cholesterol via scavenger receptors, and become foam cells. At the same time, T cells become activated and produce pro-inflammatory cytokines that further the progression of this disease[3,4]. Adaptive autoimmune responses against plaque antigens orchestrate plaque inflammation in focal lesions. It has been reported that Th1-type cytokines IFN-γ and TNF-α are pro-atherogenic and Th2- and Treg-type cytokines IL-10 and TGF-β are athero-protective, while Th17-type cytokines, IL-17A and IL-22, have controversial roles in AS[5].

For many years, CD4^+^ T cells were the focus of interest because they are the predominant T cell type in mouse atherosclerotic lesions[6]. While the role of CD8^+^ T cells in AS has been less investigated. It was recently shown that advanced human atherosclerotic plaques contain activated CD8^+^ T cells [7,8]. However, studies measuring the role of CD8^+^ T cells in AS have shown contradictory results. CD8^+^ T cells have been shown to be more frequent in early lesions and to have anti-atherogenic effects [9,10]. CD8^+^ T cells have also been shown to be important in fully established atherosclerotic plaques [11] and to have pro-atherogenic effects [12]. While some researchers have thought that CD8^+^ T cells have a minor role in the progression of AS [13,14]. Recently more and more evidence has showed that different subsets of CD8^+^ T cells have different effects on the development of AS [15].

Co-inhibitory molecules are important regulators of CD8^+^ T responses in a variety of disease conditions. Among these molecules, programmed cell death-1 (PD-1) and T cell immunoglobulin and mucin domain 3 (Tim-3) have attracted the most attention. PD-1 was identified as a marker for T cell exhaustion, and blockade of PD-1 signaling in most cases has shown to revert the dysfunctional state of exhausted CD8^+^ T cells [16,17]. Tim-3 is similar to PD-1 in its role as a negative regulator of CD8^+^ T cells function, and Tim-3 blockade can restore proliferation and cytokine production of Tim-3^+^ CD8^+^ T cells [18,19]. The co-expression of PD-1 and Tim-3 on CD8^+^ T cells identifies a most severely exhausted CD8^+^ T cell subset, and combined blockade of PD-1 and Tim-3 pathways has been shown to be the most powerful way to restore the function of exhausted CD8^+^ T cells [20,21]. Only few studies have shown that single PD-1 or Tim-3 pathway signaling has anti-atherogenic effects during the progress of murine AS [22,23,24].

To our knowledge, the functional regulation of PD-1 and Tim-3 on CD8^+^ T cells in human AS has not been previously explored. We hypothesized that co-expression of PD-1 and Tim-3 would demarcate particularly regulatory CD8^+^ T cells associated with AS. To test this hypothesis, we investigated PD-1 and Tim-3 expression on lymphocytes in the context of AS. In particular, we used detailed surface and intracellular phenotype analyses as well as multifunctional assays to study the role of Tim-3 and PD-1 signaling pathways in regulating CD8^+^ T cell function in AS.

## Materials and Methods

### Human Samples

The study group comprised 28 healthy people (age-, sex- and smoking status-matched, Table 1) and 56 patients with an atherosclerotic stenosis associated with a history of intermittent claudication or pain at rest. B-mode ultrasonography was firstly used in the evaluation of all the healthy volunteers and patients with (plaque in the femoral artery) or without (no plaque in artery) atherosclerosis. Atherosclerosis was documented angiographically in all patients. Femoral artery blood and elbow venous blood were collected after angiography. Moreover, all patients were diagnosed AS for the first time and had not received drug (except for heparin) or surgery treatment before sampling. The patients then went through percutaneous transluminal angioplasty or cutting balloon angioplasty treatment arms based on their occlusion. Eighteen of the healthy volunteers has signed the agreements to collect their femoral artery blood and elbow venous blood after angiography. Ankle-brachial index (ABI) measurements were performed before the initiation of the study.

**Table 1:** Clinical characteristics of enrolled subjects.

Subjects	AS(n = 56)	Health(n = 28)
Age mean(years)	64.94±2.16	64.17±3.33
Age range(years)	45–81	47–80
Male/female	33/23	16/12
Treatment history	-	-
Smoking period (years)	16.8±3.86	15.53±4.76
Cho(mmol/L)^a^	4.51±0.29	4.2±0.17
TG(mmol/L)^a^	1.39±0.17	1.23±0.24
HDL-C(mmol/L)^a^	1.29±0.07	1.48±0.09
LDL-C(mmol/L)^a^	2.64±0.19	2.38±0.11

Patients with atherosclerosis (AS); healthy individuals (Health); cholesterol (Cho); triglyceride (TG); high density lipoprotein cholesterol (HDL-C); low densitylipoprotein cholesterol (HDL-C)

^a^ Median±SEM.

### Ethics Statement

This study was approved by the Human Research Ethics Committee of Xinhua Hospital affiliated with Shanghai Jiaotong University School of Medicine. Every participant signed a written informed consent form.

### Cell Isolation

Blood mononuclear cells were isolated from samples of the femoral artery and peripheral blood of AS patients and healthy individuals. Whole blood containing acid citrate dextrose anticoagulant (Biological Specialty Corporation, Colmar, PA, USA) was carefully layered over an equal volume of Ficoll (Huajing, Shanghai, ρ = 1.077±0.002 g/ml) density gradient medium. Tubes containing the blood and Ficoll were centrifuged for 20 min at 2000 rpm. Isolated arterial blood mononuclear cells (ABMCs) and peripheral blood mononuclear cells (PBMCs) were washed two times in phosphate-buffered saline (PBS) and centrifuged for 10 min at 1200 rpm, and cultured in RPMI 1640 supplemented with 10% heat-inactivated fetal bovine serum (FBS; Gibco, Grand Island, NY), 100 U/ml penicillin, 100 μg/ml streptomycin, and 1μg/ml amphotericin B at 37°C in 95% air and 5% CO_2_. CD8^+^ T cells were isolated by magnetic affinity cell sorting using CD8 microbeads (Miltenyi Biotec) with LS columns following the manufacturer’s instructions. CD8^+^T purity was 96.8±7.8% as determined by flow cytometry (FCM) with FITC-CD8.

### Cell Treatment

Arterial CD8^+^ T cells were cultured (5×10^5^ per well) in the presence of 10 μg/ml anti-Tim-3 (clone F38-2E2, BioLegend), anti-PD-L1 (clone 29E.2A3, BioLegend), anti-Tim-3 plus anti-PD-L1, or isotype control. After 48 h, the culture supernatant was collected and further analyzed by FCM.

For intracellular cytokine analysis, brefeldin A (a Golgi inhibitor) (10 mg/ml, Biolegend, USA) was used for 4h (at the end of the culture) to block the secretion of cytokines into the media after the activation of cells by phorbol 12-myrstate 13-acetate (PMA) (50 ng/ml, 12h) and ionomycin (1μg/ml, 12h). Cells were harvested and analyzed by FCM for intracellular cytokine production.

### Flow Cytometry

Cell surface molecular expression and intracellular cytokine production were evaluated using FCM. FITC-conjugated anti-human CD8, CD11c, or CD3, PE-conjugated anti-human Tim-3 or GATA-3, PE/CY7-conjugated anti-human CD4, CD14, CD16, CD69, CD45RO, CD62L, IL-10, TNF-α or TGF-β, APC-conjugated anti-human PD-1 or IFN-γ, Brilliant Violet 421-conjugated anti-human CD127, CD44, IL-4, T-bet or Ki67 (Biolegend, USA) were used. For intracellular staining, cells were fixed and permeabilized using a Fix/Perm kit (Biolegend, USA). FCM analysis was performed on a Beckman-Coulter CyAN ADP flow cytometer and analyzed with FlowJo software (Tree Star, Ashland, OR, USA).

### Statistical Analysis

The significance of differences between two groups was determined by the post hoc Dunnett t test. Multiple groups were analyzed with GraphPad Prism version 5 by one-way or two-way ANOVA with Bonferroni post hoc t tests. For all statistical tests, a p value <0.05 was considered statistically significant.

## Results

### PD-1 and Tim-3 Co-Expression on CD8^+^ T Cells in AS

**Fig 1:**
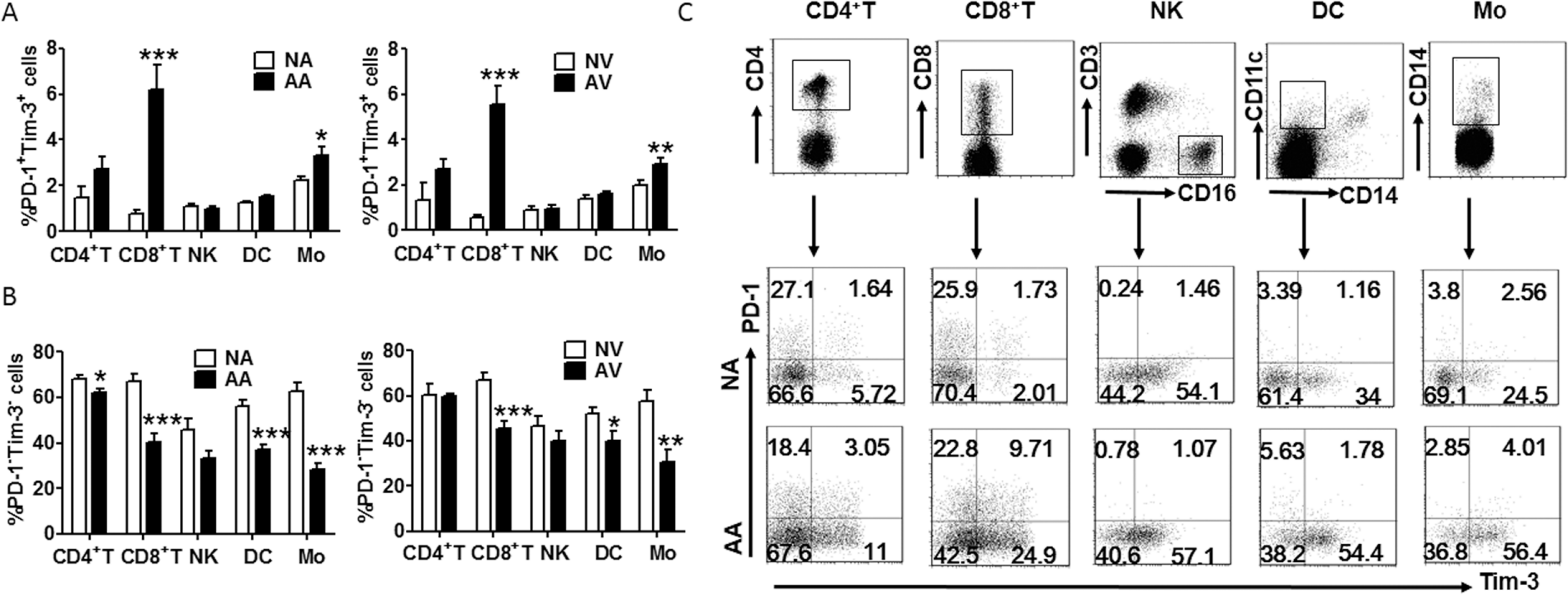
PD-1 and Tim-3 expression on CD8^+^ T cells. (A) Frequency of PD-1 and Tim-3 co-expression on different subsets of lymphocytes from lesional arterial blood and peripheral venous blood of AS patients (arterial blood of AS, AA, and venous blood of AS, AV, n = 22) and healthy individuals (normal arterial blood, NA, and normal venous blood, NV, n = 18). (B) The percentage of PD-1^**-**^ Tim-3^**-**^ cells within different cell populations in the circulation of AS patients and healthy individuals (AA, AV = 22, NA, NV = 18). (C) Representative flow cytometric dot plot showing PD-1 and Tim-3 expression on CD4^**+**^ T cells, CD8 ^**+**^ T cells, NK cells, DCs, and monocytes from NA and AA. Data represent mean ± standard error of the mean (SEM). *P<0.05; ** P<0.01; *** P<0.001.

First we certificated the existence of PD-1 and Tim-3 on arterial and peripheral lymphocytes in AS patients and healthy controls by FCM. As shown in Fig 1, Tim-3 and PD-1 expression was elevated on several immune cells both in lesional arterial and peripheral venous blood, while PD-1^-^ Tim-3^-^ cells were decreased in AS patients. Among these immune cells, CD8^+^ T cells demonstrated the most significant up-regulation of Tim-3 and PD-1. We also compared PD-1 and Tim-3 expression on arterial and peripheral blood lymphocytes form the same donors, and found that the PD-1 and Tim-3 expression levels were accordant in blood form local artery and peripheral vein (Fig 1).

### PD-1^+^ Tim-3^+^ CD8^+^ T Cell Characterization in AS

**Fig 2:**
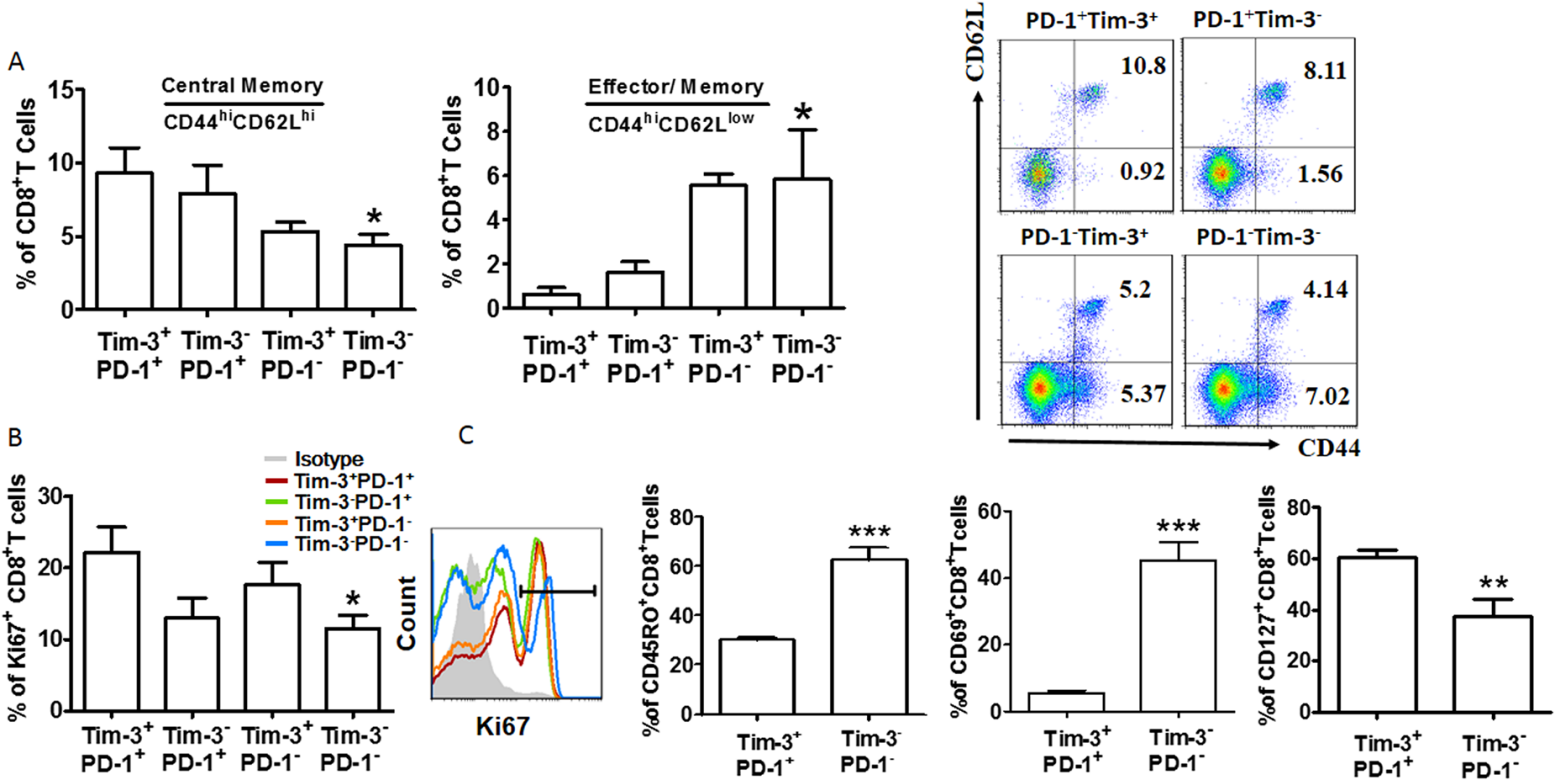
CD8^+^ T cells co-expressing Tim-3 and PD-1 display a T_CM_ phenotype in AS. (A) CD8^**+**^ T cells from donors (n = 12) were stained with antibodies against CD44 and CD62L to determine their differentiation phenotype (see Results). Percent of PD-1^**+**^ Tim-3^**+**^, PD-1^**+**^ Tim-3^**-**^, PD-1^**-**^ Tim-3^**+**^, and PD-1^**-**^ Tim-3^**-**^ cells within each population. The flow cytometry plot is from one representative experiment. (B) Quantification of Ki67 staining in PD-1^**+**^ Tim-3^**+**^, PD-1^**+**^ Tim-3^**-**^, PD-1^**-**^ Tim-3^**+**^, and PD-1^**-**^ Tim-3^**-**^ CD8^**+**^ T cells; n = 10. (C) CD8^**+**^ T cells from lesional arterial blood were stained with antibodies against PD-1, Tim-3, CD45RO, CD69, and CD127, and the PD-1^**+**^ Tim-3^**+**^ and PD-1^**-**^ Tim3^**-**^ phenotypes were compared (n = 11). Horizontal bars denote means. *P<0.05; ** P<0.01; *** P<0.001, compared with the PD-1^**+**^ Tim-3^**+**^ group.

To further characterize the different subsets of CD8^+^ T cells in lesional arteries, we first examined their expression of CD44 and CD62L. According to the different expression of these two molecules, T cells can be classified as central memory (T_CM,_ CD44^+^CD62L^+^) and effector memory (T_EM_, CD44^+^CD62L^-^)[25]. We found that the pattern of CD44 and CD62L expression was quite different among the PD-1^+^ Tim-3^+^, PD-1^+^ Tim-3^-^, PD-1^-^ Tim-3^+^, and PD-1^-^ Tim-3^-^ CD8^+^ T cells in AS. The PD-1^+^ Tim-3^+^ population contained the largest fraction of central memory cells, but the lowest fraction of effector memory cells (Fig 2A). Ki-67 is generally used as a marker of proliferating cells [26]. As seen in Fig 2B, the majority of the expanded population of CD8^+^T cells was Tim-3^+^ PD-1^+^, while the proliferation of Tim-3^-^ PD-1^-^ CD8^+^ T cells was restricted in AS. We next examined the expression of CD69, CD45RO and CD127 to determine whether CD8^+^ T cells function varied with regard to PD-1 and Tim-3 expression in AS. This investigation showed that PD-1^+^ Tim-3^+^ CD8^+^ T cells expressed lower levels of activation (CD69) and memory markers (CD45RO) but higher CD127 than PD-1^-^Tim-3^-^T cells (Fig 2C). But co-expression of PD-1 and Tim-3 on CD8^+^T cells demarcates a T_EM_ phenotypic signature in healthy individuals. Furthermore, in healthy people, PD-1^+^ Tim-3^+^ CD8^+^ T cells expressed more CD45RO and CD69 but lower Ki-67 or CD127 (S1 Fig).

### The Function of CD8^+^ T Cells Expressing PD-1 and Tim-3 in AS

**Fig 3:**
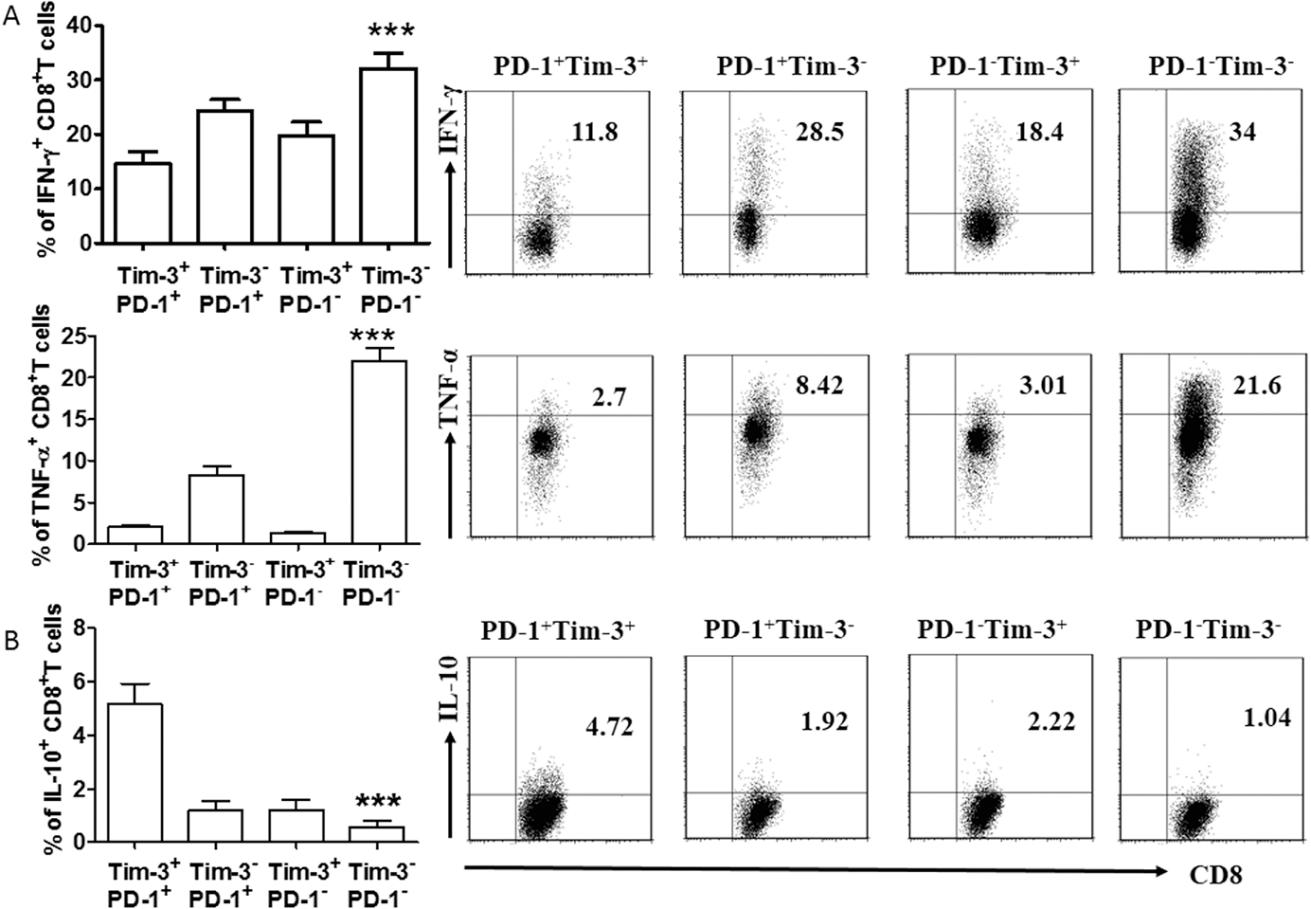
Cytokine production in CD8^+^ T cells in AS. (A) Flow cytometric analysis (right) and quantification (left) of flow cytometric analysis of pro-atherogenic cytokines (IFN-γ and TNF-α) in PD-1^**+**^ Tim-3^**+**^, PD-1^**+**^ Tim-3^**-**^, PD-1^**-**^ Tim-3^**+**^, and PD-1^**-**^ Tim-3^**-**^ CD8^**+**^ T cells (n = 12). (B) Expression of IL-10 on Tim-3^**+**^ PD-1^**+**^, Tim-3^**-**^ PD-1^**+**^, Tim-3^**+**^ PD-1^**-**^, and Tim-3^**-**^ PD-1^**-**^ CD8^**+**^ T cells in AS. Flow cytometric analysis (right) and quantitation (left); n = 17. Data represent mean ± SEM. Flow cytometry plot is from one representative experiment. ** P<0.01; ***P<0.001, compared with the PD-1^**+**^ Tim-3^**+**^ group.

To determine whether any of the Tim-3- and PD-1-expressing CD8^+^ T cells in AS exhibited an anti-atherogenic phenotype, fresh isolated ABMCs from local lesional arteries were stimulated with PMA/Ionocymin for 12 h, then stained for CD8 expression, and finally examined for production of pro-atherogenic cytokines (INF-γ and TNF-α) and an anti-atherogenic cytokines (IL-10). We found that PD-1^+^ Tim-3^+^ CD8^+^ T cells are associated with decreased pro-atherogenic cytokines but increased anti-atherogenic cytokines (Fig 3A and 3B). We also observed more IL-4 production in PD-1^+^ Tim-3^+^ CD8^+^ T cells (S2A Fig). However, there was no difference in TGF-β production (S2B Fig). Interestingly, PD-1^-^ Tim-3^+^ CD8^+^ T and PD-1^+^ Tim-3^-^ CD8^+^ T cells produced more anti-atherogenic but less pro-atherogenic cytokines than Tim-3^-^ PD-1^-^ CD8^+^ T cells, which further underlines the important roles of Tim-3 and PD-1 may play in the regulation of CD8^+^ T cells function in AS.

### Effects of Targeting Tim-3 and PD-1 Signaling Pathways on CD8^+^ T Cells in AS

**Fig 4:**
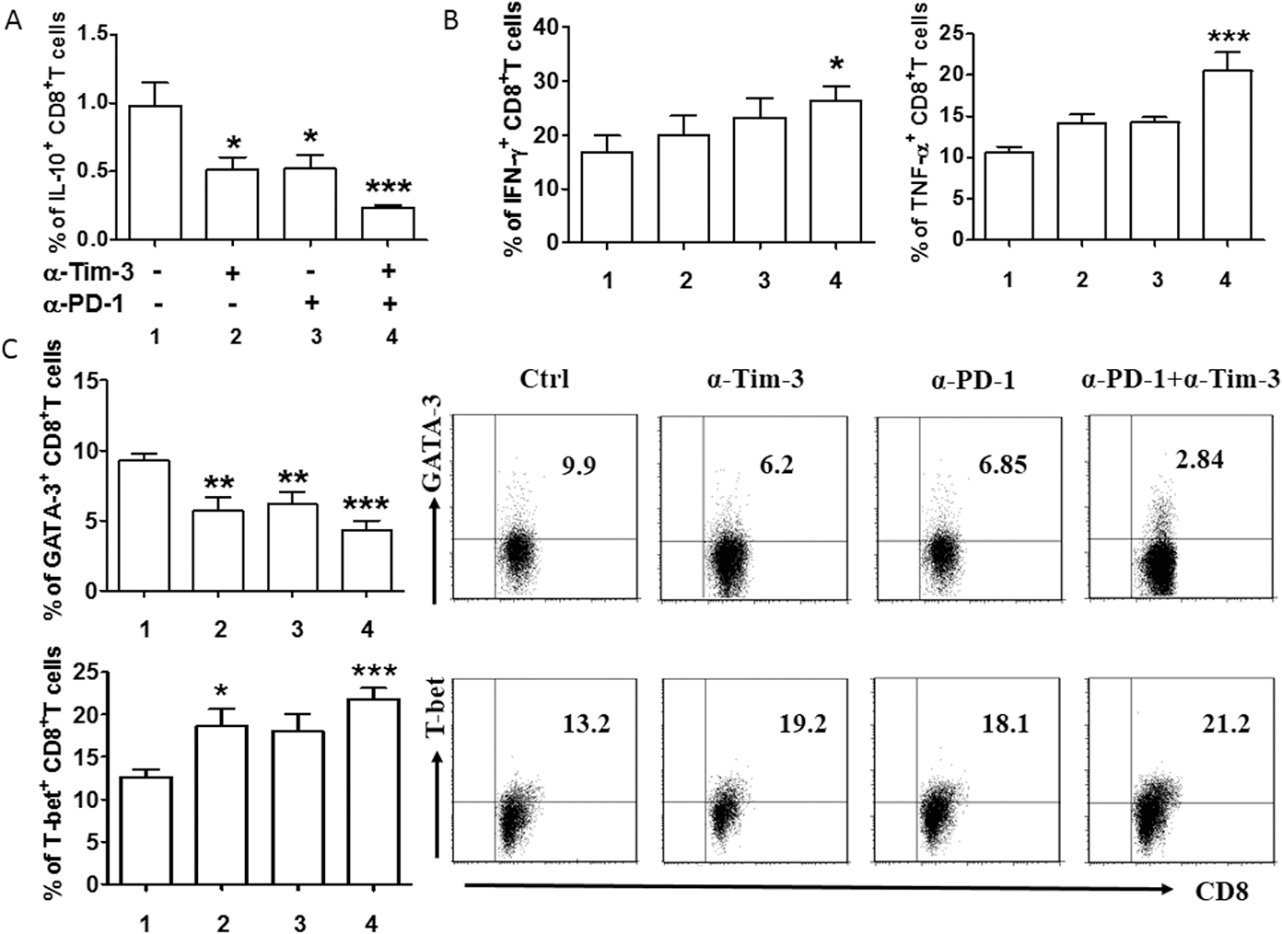
Effect of targeting Tim-3 and PD-1 signaling pathways on CD8^+^ T cells function. (A and B) Quantification of flow cytometric analysis of cytokine production by different subsets of CD8^**+**^ T cells cultured for 48 h in the presence or absence of anti-Tim-3 antibody (10 μg/ml), anti-PD-L1 antibodies (10 μg/ml), or both anti-Tim-3 and anti-PD-L1. (C) Quantification (left) and the representative figures of flow cytometric analysis (right) of transcription factors by CD8^**+**^ T cells following treatment with the indicated blocking antibodies. Data represent mean ± SEM (n = 16). CD8^**+**^ T cells from the lesional artery. *P<0.05, **P<0.01, ***P<0.001, compared with the control group.

As noted previously, individual or combined blockade of Tim-3 and PD-1 can restore the function of exhausted CD8^+^ T cells. We explored the effects of single or dual blockade of PD-1 and Tim-3 on the cytokine production of CD8^+^ T cells and the immune microenvironment at the lesion site. CD8^+^T cells from local lesion sites were stimulated with PMA/Ionomycin alone, PMA/Ionomycin plus anti-Tim-3 blocking antibody, PMA/Ionomycin plus anti-PD-L1 blocking antibody, or PMA/Ionomycin plus blocking antibodies to both PD-L1 and Tim-3. We found that both anti-Tim-3 and anti-PD-L1 alone were able to reduce IL-10 production of CD8^+^ T cells. However, this effect was weaker when compared with the decrease in IL-10 production of CD8^+^ T cells treated with both anti-Tim-3 and anti-PD-L1 antibodies (Fig 4A). A similar phenomena was observed in the production of another Th2 cytokine, IL-4, while the blockade had no effect on TGF-β generation (S3 Fig). Meanwhile, we observed an increase in INF-γ and TNF-α production after combined blockade of Tim-3 and PD-1 (Fig 4B). To validate the decrease IL-10 and IL-4 production by CD8^+^ T cells, we tested the expression of Th2-type key transcription factor-GATA-3 (since IL-4 and IL-10 are also known as Th2-type cytokines)[27]. We found a significant reduction in the expression of GATA-3 in CD8^+^ T cells following single or combined antibody blockade (Fig 4C). We also examined the Th1-specific transcription factor-T-bet (since INF-γ and TNF-α are also known as Th1-type cytokines) and found increased expression of T-bet, in accordance with increased production of Th1-type cytokines (Fig 4C).

## Discussion

In the present study, we verified the expression of PD-1 and Tim-3 on CD8^+^ T cells in AS both within the lesional arterial and peripheral venous lymphocytes. Interestingly, the dual expression of PD-1 and Tim-3 on CD8^+^ T from AS patients was higher than that on CD8^+^ T cells from healthy individuals both in local arterial and peripheral venous cells, while PD-1^-^ Tim-3^-^ CD8^+^ T cells were less abundant in AS. Notably, the co-expression of PD-1 and Tim-3 was associated with T_CM_ phenotype and increased anti-atherogenic cytokine production but decreased pro-atherogenic cytokine production by CD8^+^ T cells from the lesional arterial blood. We further showed that targeting PD-1 and Tim-3 signaling pathways in CD8^+^ T cells resulted in a decrease in anti-atherogenic cytokine production and in an augmentation in the generation of pro-atherogenic cytokines. These findings, for the first time, highlight the important roles that PD-1 and Tim-3 signaling pathways may play in the regulation of CD8^+^ T cells function in AS.

The role of CD8^+^ T cells in AS remains unclear, since contradictory effects of this cell on AS have been reported[10,12,13], including pro-atherogenic, anti-atherogenic, or no influence on AS. The idea that different subsets of CD8^+^ T cells have different effects on AS may explain this paradox. Although CD8^+^ effector cytotoxic T lymphocytes promote the development of AS[7,12], the adoptive transfer of CD8^+^ CD25^+^ T cells has been shown to reduce atherosclerotic lesions[28]. Furthermore, CD8^+^ CD28^high^ and CD8^+^ CD25^+^ T cells can reduce neointima formation after arterial injury [29,30]. PD-1^+^ Tim-3^+^ CD8^+^ T cells are the most severely exhausted CD8^+^ T cell subset, and combined blockade of ligand-receptor interactions by PD-1 and Tim-3 can restore the function of exhausted CD8^+^ T cells[20,21]. The predominance of CD8^+^ T cells in human atherosclerotic lesions [8], the “regulatory” CD8^+^ T cell athero-protective effects, and PD-1^+^ Tim-3^+^ CD8^+^ T cell “exhausted” phenotype, led us to investigate this particular T-lymphocyte subset in human AS.

PD-1 is a member of B7 family on T cells [31]. Although it has been reported that Tim-3 is mainly expressed on NK cells, DCs, and monocytes during AS development in LDLr^-/-^mice [22,32], the co-expression of PD-1 and Tim-3 was up-regulated mainly on CD8^+^ T cells in AS both in lesional arterial and peripheral venous blood. So we wondered whether the Tim-3 and PD-1 signaling pathways are involved in the regulation of CD8^+^ T cell function in AS. Based on the variable expression of CD44 and CD62L, CD8^+^ T cells can be divided into two subsets, T_CM_ (CD44^+^CD62L^+^) and T_EM_ (CD44^+^CD62L^-^)[25]. We found that, in AS, the largest fraction of CD8^+^ T_CM_ cells but the lowest fraction of CD8^+^T_EM_ cells were PD-1^+^ Tim-3^+^, while the largest fraction of CD8^+^ T_EM_ cells but the lowest fraction of CD8^+^ T_CM_ cells were PD-1^-^ Tim-3^-^. CD8^+^ T_CM_ cells are less cytolytic and exhibit increased ability of self-renewal compared to CD8^+^ T_EM_ cells[33,34,35]. Consistent with this, PD-1^+^ Tim-3^+^ CD8^+^ T cells proliferated more than PD-1^-^ Tim-3^-^ CD8^+^ T cells. This is contrary to what has been observed in chronic viral infection[36].We speculated that this may be due to the somewhat divergent phenotypes of PD-1^+^ Tim-3^+^ CD8^+^ T cells in AS. Further phenotypic profiling demonstrated that these cells expressed less activation and memory markers but higher CD127 than PD-1^-^Tim-3^-^CD8^+^ T cells. CD127 is well known to be lost in exhausted T cells during chronic viral infection[37]. But we observed the opposed results in healthy people. Together with the higher proliferation of PD-1^+^ Tim-3^+^ CD8^+^ T cells, we presume that the co-expression of PD-1 and Tim-3 on CD8^+^ T cells in AS could not be simply regarded as an “exhausted” marker. Further research in needed to explore the exact mechanism that formation the unique phenotype of PD-1^+^Tim-3^+^CD8^+^T cells in AS.

Chronic inflammation is an important factor during the development of AS. Pro-inflammatory cytokines such as IFN-γ and TNF-α can enhance lesion development, while anti-inflammatory cytokines such as IL-10 have a protective role in AS[38]. Double-positive PD-1^+^ and Tim-3^+^ CD8^+^ T cells generate more anti-atherogenic cytokines (IL-10, also known as Th2-type cytokines) and less pro-atherogenic cytokines (IFN-γ and TNF-α, also known as Th1-type cytokines) than single or negative CD8^+^ T cells. Single targeting of the Tim-3 and PD-1 pathways has variable effects on the function of CD8^+^ T cells from the lesional arterial blood, whereas combined targeting of these pathways is highly effective in enhancing pro-atherogenic cytokine and reducing anti-atherogenic cytokine production by PD-1^+^ Tim-3^+^ CD8^+^ T cells from the lesional arterial blood, as well as their respective transcription factors but not affects cells viability (data not shown). Therefore, we hypothesize that higher expression levels of PD-1 and Tim-3 is a physiological response to the atherosclerotic environment. The expression of Tim-3 and PD-1 by CD8^+^ T cells may be important, at least in part for preservation of AS from deteriorating. However, whether it is the atherosclerotic microenvironment that shapes the distinct CD8^+^ T subset and that which differentiates CD8^+^ T cells in this specific microenvironment still needs to be further determined.

We also observed more IL-4 production by PD-1^+^ Tim-3^+^ CD8^+^T cells, and that blockage of Tim-3 and PD-1 pathways can suppress the generation of IL-4. While the exact role of IL-4 in AS is still unclear[38], given its anti-inflammatory and Th1-inhibitory potency[39], we suspect that the production of IL-4 by Tim-3^+^ PD-1^+^ CD8^+^ T cells plays a similar role as does IL-10. TGF-β is a potent anti-inflammatory and anti-atherogenic cytokine[38], but we did not see differences in its production among different subsets of CD8^+^ T cells. In addition, interference of PD-1 and Tim-3 signaling also has no effect on its production. It has been reported that TGF-β has an opposite effect on PD-1 and Tim-3 expression[40]; however, whether PD-1 and Tim-3 also have opposite effects on TGF-β expression and whether the joint participation of these two molecules has any effect on TGF-β expression still needs further study.

Our data demonstrate, for the first time to our knowledge, that the co-expression of PD-1 and Tim-3 on CD8^+^ T is up-regulated in human AS, and that this distinct CD8^+^ T cell subset cannot be simply regarded as an exhausted subset. PD-1^+^ Tim-3^+^ CD8^+^ T cells from the lesional arterial blood produced more anti-atherogenic cytokines, and blockade of PD-1 and Tim-3 signaling pathways aggravated the pro-inflammatory Th1 responses predominate in AS. This knowledge should be considered to add further information regarding risk of AS since PD-1 and Tim-3 are the targets of new therapies for chronic viral infections. Moreover, since single blockade of PD-1 and Tim-3 has been shown to augment atherosclerotic lesion development in experimental AS[22,23,24], approaches to promote the PD-1 and Tim-3 pathway may represent novel therapeutic strategies to inhibiting atherosclerotic lesion development and preventing cardiovascular disease.

## Supporting Information

S1 Fig CD8^+^ T cells co-expressing Tim-3 and PD-1 display a T_EM_ phenotype in healthy people. (A) CD8^+^ T cells from healthy individuals (NCD8^+^T) (n = 10) were stained with antibodies against CD44 and CD62L to determine their differentiation phenotype (see Results). Percent of PD-1^+^ Tim-3^+^ and PD-1^-^Tim-3^-^cells within each population. (B) Quantification of Ki67 staining in PD-1^+^ Tim-3^+^ and PD-1^-^ Tim-3^-^ CD8^+^ T cells; n = 10. (C) CD8^+^ T cells from venous blood of healthy individuals were stained with antibodies against PD-1, Tim-3, CD45RO, CD69, and CD127, and the PD-1^+^ Tim-3^+^ and PD-1^-^Tim3^-^phenotypes were compared (n = 10). Horizontal bars denote means. *P<0.05; *** P<0.001, compared with the PD-1^+^ Tim-3^+^ group.
(PDF)

S2 Fig Cytokine production in CD8^+^ T cells during AS. Quantification of flow cytometric analysis of IL-4 (A) and TGF-β (B) in PD-1^+^ Tim-3^+^, PD-1^+^ Tim-3^-^, PD-1^-^ Tim-3^+^, and PD-1^-^ Tim-3^-^ CD8^+^ T cells in AS; n = 18. Data represent mean ± SEM. * P<0.05; compared with the PD-1^+^ Tim-3^+^ group.
(PDF)

S3 Fig Cytokine production by CD8^+^ T cells after targeting Tim-3 and PD-1 signaling pathways. Quantification of flow cytometric analysis of IL-4 (left) and TGF-β (right) production by CD8^+^ T cells cultured for 48 h in the presence or absence of anti-Tim-3 antibody (10 μg/ml), anti-PD-L1 antibodies (10 μg/ml), or both anti-Tim-3 and anti-PD-L1. Data represent mean ± SEM (n = 16). CD8^+^ T cells from the lesional artery. *P<0.05, compared with the control group.
(PDF)
