# Supplementary figures and images for: PD-1 and Tim-3 Pathways Regulate CD8+ T Cells Function in Atherosclerosis
Source: PLoS One. 2015 Jun 2;10(6):e0128523. doi: 10.1371/journal.pone.0128523 (PMC4452700; doi:10.1371/journal.pone.0128523)

Figure S1

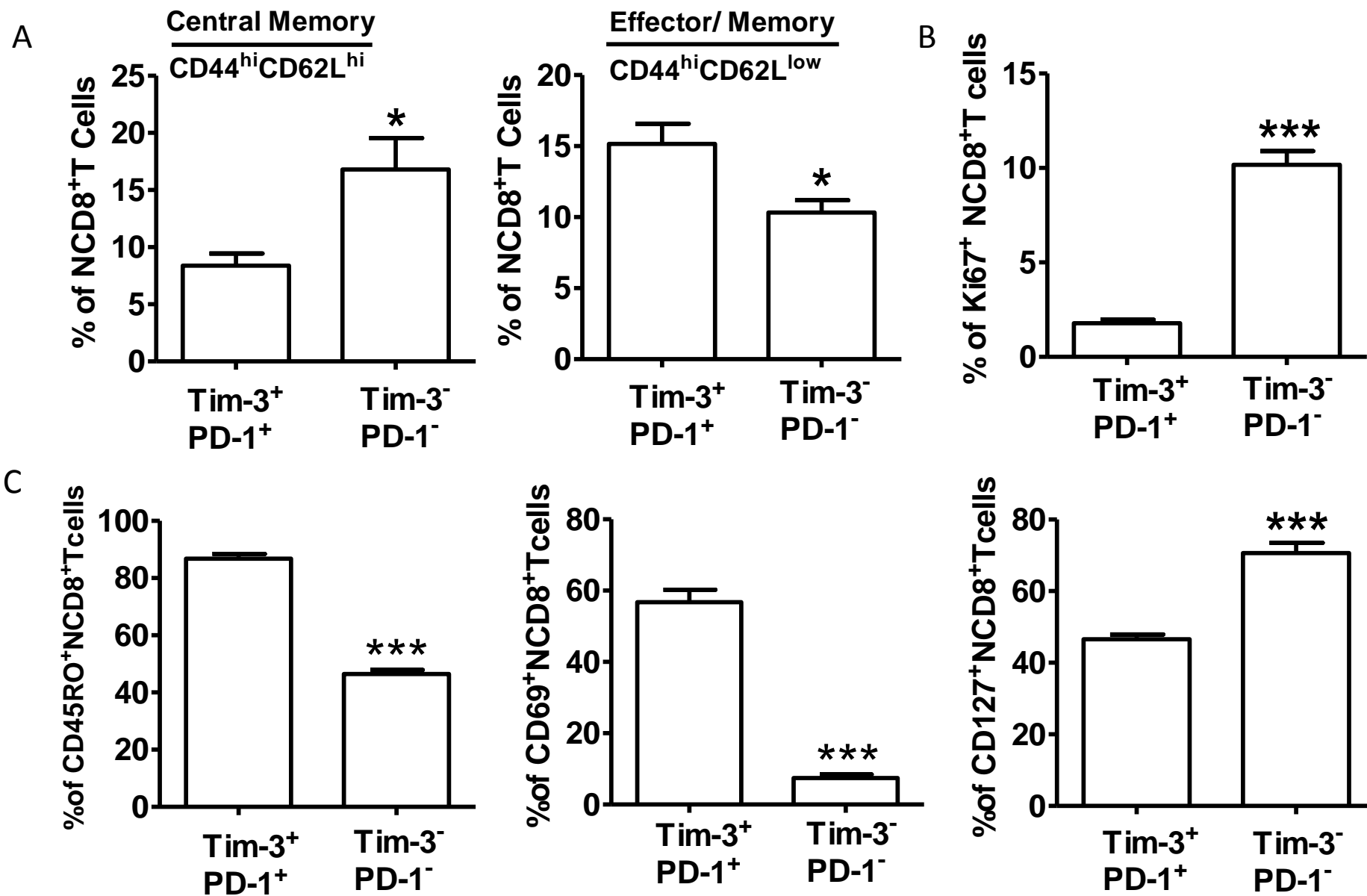

Supplement: S1 Fig — (A) CD8+ T cells from healthy individuals (NCD8+T) (n = 10) were stained with antibodies against CD44 and CD62L to determine their differentiation phenotype (see Results). Percent of PD-1+ Tim-3+ and PD-1-Tim-3-cells within each population. (B) Quantification of Ki67 staining in PD-1+ Tim-3+ and PD-1- Tim-3- CD8+ T cells; n = 10. (C) CD8+ T cells from venous blood of healthy individuals were stained with antibodies against PD-1, Tim-3, CD45RO, CD69, and CD127, and the PD-1+ Tim-3+ and PD-1-Tim3-phenotypes were compared (n = 10). Horizontal bars denote means. *P<0.05; *** P<0.001, compared with the PD-1+ Tim-3+ group. (PDF) [file pone.0128523.s001.pdf]

Figure S2

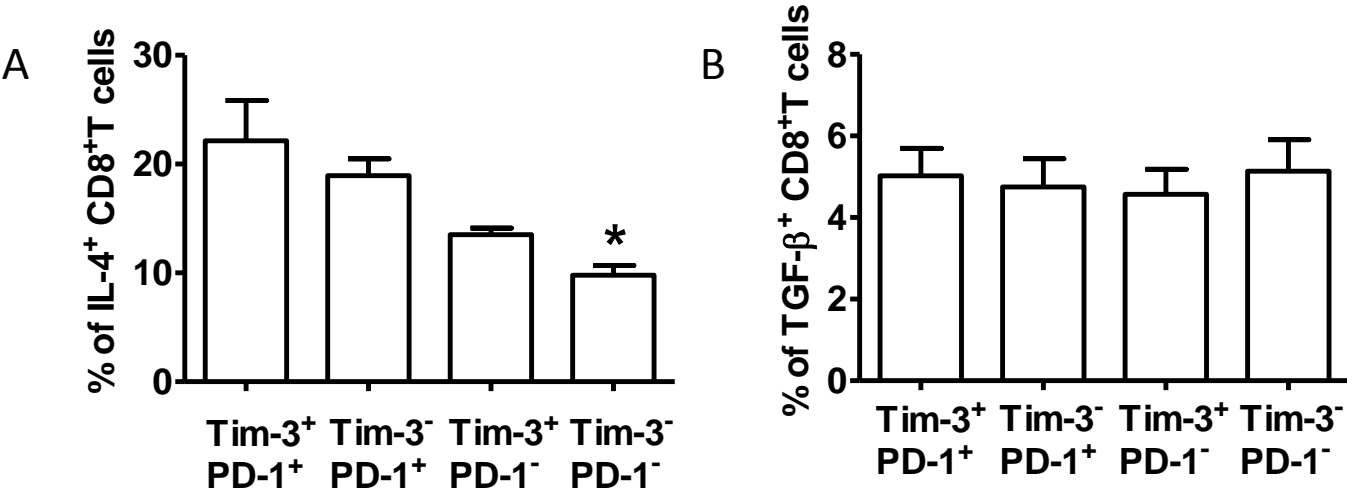

Supplement: S2 Fig — Quantification of flow cytometric analysis of IL-4 (A) and TGF-β (B) in PD-1+ Tim-3+, PD-1+ Tim-3-, PD-1- Tim-3+, and PD-1- Tim-3- CD8+ T cells in AS; n = 18. Data represent mean ± SEM. * P<0.05; compared with the PD-1+ Tim-3+ group. (PDF) [file pone.0128523.s002.pdf]

Figure S3

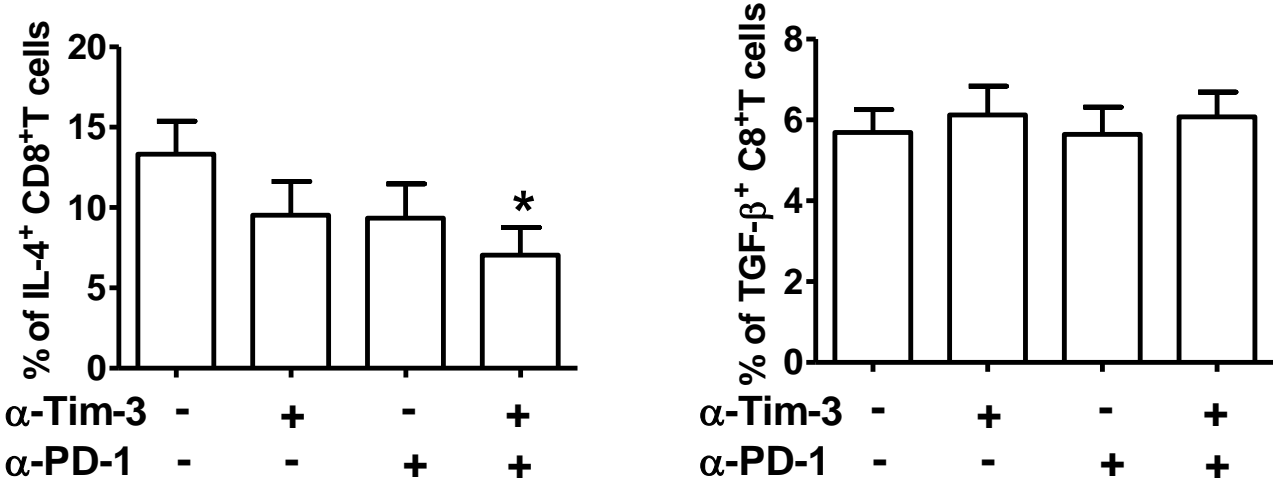

Supplement: S3 Fig — Quantification of flow cytometric analysis of IL-4 (left) and TGF-β (right) production by CD8+ T cells cultured for 48 h in the presence or absence of anti-Tim-3 antibody (10 μg/ml), anti-PD-L1 antibodies (10 μg/ml), or both anti-Tim-3 and anti-PD-L1. Data represent mean ± SEM (n = 16). CD8+ T cells from the lesional artery. *P<0.05, compared with the control group. (PDF) [file pone.0128523.s003.pdf]
